# Supplementary material for: Nonregistration, discontinuation, and nonpublication of randomized trials: A repeated metaresearch analysis
Source: PLoS Med. 2022 Apr 27;19(4):e1003980. doi: 10.1371/journal.pmed.1003980 (PMC9094518; doi:10.1371/journal.pmed.1003980)
Supplement: S1 Text — (DOCX) [file pmed.1003980.s002.docx]

**S1 Text: Information on all participating research ethics committees**

**Switzerland**:

**Basel**: Ethikkommission Nordwest- und Zentralschweiz (EKNZ), Hebelstrasse 53, 4056 Basel

**Bellinzona:** Comitato etico cantonale, c/o Ufficio di sanità, Via Orico 5, 6501 Bellinzona

**Bern**: Kantonale Ethikkommission Bern, Murtenstrasse 31, 3010 Bern

**Geneva**: Ethics Committee Geneva, Rue Adrien-Lachenal 8, 1207 Genève

**Lausanne**: Ethics Committee Vaud, Avenue de Chailly 23, 1012 Lausanne

**St. Gallen**: Ethikkommission Ostschweiz, Oberer Graben 32, 9001 St.Gallen

**Thurgau**: Has joined the «Ethikkommission Ostschweiz” in 2016. See therefore details for “St. Gallen”

**United Kingdom:**

**Bristol office of the UK National Research Ethics Service**: Health Research Authority, Temple Quay House, 2 The Square, Temple Quay, Bristol BS1 6PN

**Germany:**

**Freiburg**: Ethik-Kommission der Albert-Ludwigs-Universität Freiburg, Hugstetter Strasse 55, 79106 Freiburg

**Canada:**

**Hamilton:** Hamilton Integrated Research Ethics Board (HiREB), 293 Wellington Street North, Suite 102, Hamilton Ontario, L8L 8E7
