# Supplementary material for: Nonregistration, discontinuation, and nonpublication of randomized trials: A repeated metaresearch analysis
Source: PLoS Med. 2022 Apr 27;19(4):e1003980. doi: 10.1371/journal.pmed.1003980 (PMC9094518; doi:10.1371/journal.pmed.1003980)
Supplement: S2 Text — (DOCX) [file pmed.1003980.s003.docx]

**S2 Text: Procedure to receive more information about the trial status from ethical committees or by contacting principal investigators by sending them a survey through ethical committees**

**Switzerland**: For all studies for which we either (i) could not identify a trial registry entry, (ii) a full text publication, (iii) it remained unclear if the trial was discontinued (including reason for discontinuation), a survey was send to the investigator in Switzerland trough research ethical committees. A reminder was sent approximately four weeks later through our study team. The survey is presented in **S3 Text**.

**United Kingdom:** The National Health Service (NHS) Health Research Authority provided information about which trials were stopped before recruiting a first patient. For studies that were listed on the website of the NHS Health Research Authority (<https://www.hra.nhs.uk/planning-and-improving-research/application-summaries/research-summaries/>) the study team contacted the listed investigator directly (see survey **S3 Text**) if crucial information were missing (i.e. (i) could not identify a trial registry entry, (ii) a full text publication, (iii) it remained unclear if the trial was discontinued (including reason for discontinuation)).

**Germany:** The research ethical committee provided information about which trials were stopped before recruiting a first patient.

**Canada**: The research ethical committee provided information about which trials were stopped before recruiting a first patient. Investigators to which the study team had a personal contact were asked for further information in case something remained unclear ((i) could not identify a trial registry entry, (ii) a full text publication, (iii) it remained unclear if the trial was discontinued (including reason for discontinuation)).
