# Supplementary material for: Nonregistration, discontinuation, and nonpublication of randomized trials: A repeated metaresearch analysis
Source: PLoS Med. 2022 Apr 27;19(4):e1003980. doi: 10.1371/journal.pmed.1003980 (PMC9094518; doi:10.1371/journal.pmed.1003980)
Supplement: S3 Text — (DOCX) [file pmed.1003980.s004.docx]

**S3 Text:** **Survey to receive more information from investigators about the fate of their trial**

**Trial status**

1. WAS The Trial Started at all?

**YES**  **NO**  **UNKNOWN**

- - If NO, please explain why:
  - Click or tap here to enter text.

______________________________________________________________

- - If NO, check the appropriate box (← applies only to **multicenter** studies):
    - *Whole study* was not started
    - Not started only in *your center*

1. Is the Trial Registered?

**YES**  **NO**  **UNKNOWN**

- - IF **YES,** please list the registration numbers from all relevant registries:

Click or tap here to enter text.

____________________________________________________________________

1. Is the Trial still running?

**YES**  **NO**  **UNKNOWN**

- - IF **YES** (Check all that apply):
    - Recruitment not completed yet
    - Recruitment completed
    - Data collection completed

If the **trial is still running**, you can stop here and leave the rest of the **questionnaire** empty.

If the **trial is NOT running anymore**, **please proceed** with this questionnaire.

1. Has the Trial BEEN STOPPED early (before reaching the target sample as outlined in the protocol)?

**YES**  **NO**  **UNKNOWN**

- - If **YES** (Check all that apply):
    - Stopped because of slow recruitment
    - Stopped for harm
    - Stopped for benefit
    - Stopped because of evidence from other trials
    - Stopped for futility
    - Other reason (please specify)

Specification/comments:

Click or tap here to enter text.

__________________________________________________________________________

**Publication status of the trial**

1. Are the results of this trial available?

**YES**  **NO**  **UNKNOWN**

If **YES,** what was the type of the publication (check all that apply)

- - Results in **Clinical trial register**
  - Publication in **Journal**
  - **Abstract** presentation at conference
  - **Internet Report**
  - **Other form** (please specify below)

Please give **all references** of the Publication(S) with **Name of The first author**, **Title of the Publication**, **Name of the Journal/ Conference**/**other Form** and **Publication year**. For **Internet Publications** please provide the **Webaddress:**

Click or tap here to enter text.

_________________________________________________________________________________
