# Supplementary material for: Nonregistration, discontinuation, and nonpublication of randomized trials: A repeated metaresearch analysis
Source: PLoS Med. 2022 Apr 27;19(4):e1003980. doi: 10.1371/journal.pmed.1003980 (PMC9094518; doi:10.1371/journal.pmed.1003980)
Supplement: S4 Text — (DOCX) [file pmed.1003980.s005.docx]

**S4 Text:** **Search strategy to identify corresponding full text publications**

The search was conducted in the following order until a corresponding full text publication was identified.

- Publication of primary results linked in trial register?
- Search on Pubmed for ““intervention” AND “indication” using an RCT filter (also add last name of principle investigator if necessary).
- Search on Google scholar for ““intervention” AND “indication” AND “trial” (also add last name of principle investigator if necessary).
- Search on Scopus for ““intervention” AND “indication” (also add last name of principle investigator if necessary)

All searches were also conducted with and without trial acronyms whenever available.
