# Supplementary material for: Nonregistration, discontinuation, and nonpublication of randomized trials: A repeated metaresearch analysis
Source: PLoS Med. 2022 Apr 27;19(4):e1003980. doi: 10.1371/journal.pmed.1003980 (PMC9094518; doi:10.1371/journal.pmed.1003980)
Supplement: S5 Text — (DOCX) [file pmed.1003980.s006.docx]

**S5 Text:** **Code for analysis in Stata**

*Generate Results at all

generate resultsatall=0

replace resultsatall=1 if ResultsonTrialregister==1

replace resultsatall=1 if Publication==1

*Generate Registered before first patient entered (regbeforeenrollment)

generate regbeforeenrollment=.

replace regbeforeenrollment= Startearliest-Regearliestminusonemonth

replace regbeforeenrollment=1 if regbeforeenrollment>=-1

replace regbeforeenrollment=0 if regbeforeenrollment<=0

replace regbeforeenrollment=0 if Registration==0

*Generate published in journal and published in registry (publishedjournalandregister)

generate publishedjournalandregister=0

replace publishedjournalandregister=1 if ResultsonTrialregister==1 & Publication2Conferenceabstrac==1

*Generate Neither registered or results published (neitherregisterorpublished)

generate neitherregisterorpublished=1

replace neitherregisterorpublished=0 if Registration==1 | Publication2Conferenceabstrac==1

*Generate poor reccruitment and published for mulivarable analysis (poorrecruit)

generate poorrecruit=0

replace poorrecruit=1 if Reasonfordiscontinuation1rec==1

*Generate disco for preventable reason (discopreventable; reasons: (1=recruitment;2=limited resources/time constraints;3=futility;4=benefit;5=harm; 6=organisational/strategic; 7=external evidence...8=other;9=unclear)

generate discopreventable=0

replace discopreventable=1 if Reasonfordiscontinuation1rec==1

replace discopreventable=1 if Reasonfordiscontinuation1rec==2

replace discopreventable=1 if Reasonfordiscontinuation1rec==6

replace discopreventable=1 if Reasonfordiscontinuation1rec==8

replace discopreventable=1 if Reasonfordiscontinuation1rec==9

*Generate sample size /100 for ORs incremental steps and ASPIREpro*10

generate samplesize_100=samplesizeprotocolnumbers/100

generate ASPIREprop_100=ASPIREprop*10

*Generate not published (nonpublished)

gen nonpublished=0

replace nonpublished=1 if published==0

*All analysis with the following commands: “Tab”, Sum, detail”, “cii proportion”, “logistic”, “cci”

*Logistic regressions (Table 4 in manuscript and S5 in appendix)

logistic nonpublished ASPIREprop_10

logistic nonpublished samplesize_100

logistic nonpublished Placebo_yes

logistic nonpublished singlecentre_yes

logistic nonpublished Recruitpredict_n

logistic nonpublished industry

logistic nonpublished ASPIREprop_10 samplesize_100 Placebo_yes singlecentre_yes Recruitpredict_n industry

logistic poorrecruit ASPIREprop_10

logistic poorrecruit samplesize_100

logistic poorrecruit Placebo_yes

logistic poorrecruit singlecentre_yes

logistic poorrecruit industry

logistic poorrecruit Recruitpredict_n

logistic poorrecruit ASPIREprop_10 samplesize_100 Placebo_yes singlecentre_yes Recruitpredict_n industry

logistic discopreventable ASPIREprop_10

logistic discopreventable samplesize_100

logistic discopreventable Placebo_yes

logistic discopreventable singlecentre_yes

logistic discopreventable Recruitpredict_n

logistic discopreventable industry

logistic discopreventable ASPIREprop_10 samplesize_100 Placebo_yes singlecentre_yes Recruitpredict_n industry

*Association between completion of a randomised controlled trial and (Table S3 in appendix)

cci 190 66 13 32

cci 118 54 85 44

cci 198 85 5 13

cci 8 19 5 13

*Logistic regressions (Table S5 in appendix)

drop if Country=="UK"

logistic nonpublished ASPIREprop_10

logistic nonpublished samplesize_100

logistic nonpublished CTU_CRO_yes

logistic nonpublished Placebo_yes

logistic nonpublished singlecentre_yes

logistic nonpublished Recruitpredict_n

logistic nonpublished industry

logistic nonpublished ASPIREprop_10 samplesize_100 Placebo_yes singlecentre_yes Recruitpredict_n industry

logistic poorrecruit ASPIREprop_10

logistic poorrecruit samplesize_100

logistic poorrecruit CTU_CRO_yes

logistic poorrecruit Placebo_yes

logistic poorrecruit singlecentre_yes

logistic poorrecruit industry

logistic poorrecruit Recruitpredict_n

logistic poorrecruit ASPIREprop_10 samplesize_100 Placebo_yes singlecentre_yes Recruitpredict_n industry

logistic discopreventable ASPIREprop_10

logistic discopreventable samplesize_100

logistic discopreventable CTU_CRO_yes

logistic discopreventable Placebo_yes

logistic discopreventable singlecentre_yes

logistic discopreventable Recruitpredict_n

logistic discopreventable industry

logistic discopreventable ASPIREprop_10 samplesize_100 CTU_CRO_yes Placebo_yes singlecentre_yes Recruitpredict_n industry
