# Supplementary material for: Nonregistration, discontinuation, and nonpublication of randomized trials: A repeated metaresearch analysis
Source: PLoS Med. 2022 Apr 27;19(4):e1003980. doi: 10.1371/journal.pmed.1003980 (PMC9094518; doi:10.1371/journal.pmed.1003980)
Supplement: S1 Table — (DOCX) [file pmed.1003980.s007.docx]

**S1 Table: Baseline characteristics of included randomised controlled trials stratified by country of ethical approval.**

|  | **RCTs approved in Switzerland (n=165)** | **RCTs approved in the UK (n=89)** | **RCTs approved in Germany (n=37)** | **RCTs approved in Canada (n=35)** | **All RCTs (n=326)** |
| --- | --- | --- | --- | --- | --- |
| Planned sample size, median (IQR)^a^ | 250 (92, 649) | 180 (96, 450) | 172 (124, 600) | 545 (320, 1700) | 250 (100, 600) |
| Proportion of adequate SPIRIT reporting in study protocol, median (IQR) | 0.74 (0.64-0.80) | 0.65 (0.58,0.69) | 0.73 (0.66, 0.76) | 0.67 (0.60, 0.72) | 0.69 (0.61, 0.77) |
| Single centre vs. multicentre |  |  |  |  |  |
| Single centre | 34 (20.6%) | 19 (21.4%) | 5 (13.5%) | 2 (5.7%) | 60 (18.4%) |
| Multicentre | 131 (79.4%) | 70 (78.7%) | 32 (86.5%) | 33 (94.3%) | 266 (81.6%) |
| Study design |  |  |  |  |  |
| Parallel | 147 (89.1%) | 83 (93.3%) | 35 (94.6%) | 31 (88.6%) | 296 (90.8%) |
| Crossover | 9 (5.5%) | 3 (3.4%) | 1 (2.7%) | 0 (0%) | 13 (4.0%) |
| Factorial | 5 (2.2%) | 3 (3.4%) | 0 (0%) | 2 (5.7%) | 10 (3.1%) |
| Cluster | 1 (0.6%) | 0 (0%) | 1 (2.7%) | 2 (5.7%) | 4 (1.2%) |
| Other^b^ | 3 (1.8%) | 0 (0%) | 0 (0%) | 0 (0%) | 3 (0.9%) |
| Placebo controlled | 62 (37.6%) | 31 (34.8%) | 20 (54.1%) | 18 (51.4%) | 131 (40.2%) |
| Not placebo controlled | 103 (62.4%) | 58 (65.2%) | 17 (46.0%)) | 17 (48.6%) | 195 (59.8%) |
| Recruitment projection reported in protocol | 58 (35.2%) | 20 (22.5%) | 14 (37.8%) | 7 (20.0%) | 99 (30.4%) |
| No recruitment projection reported | 107 (64.9%) | 69 (77.5%) | 23 (62.2%) | 28 (80.0%) | 227 (69.6%) |
| Sponsorship |  |  |  |  |  |
| Industry | 87 (52.7%) | 45 (50.6%) | 26 (70.3%) | 21 (60.0%) | 179 (54.9%) |
| Investigator | 78 (47.3%) | 44 (49.4%) | 11 (29.7%) | 14 (40.0%) | 147 (45.1%) |

^a^ Missing data for planned sample size for 4 trial protocols was inserted from other sources (i.e. peer reviewed publication; n=3; trial registry; n=1).

^b^ Split body (n=2), parallel group with two consecutive randomisations (n=1)

Abbreviations: RCT=Randomized clinical trial; IQR=Inter quartile range; SPIRIT= Standard Protocol Items: Recommendations for Interventional Trials [1, 2]

**References:**

1. Chan AW, Tetzlaff JM, Altman DG, Laupacis A, Gotzsche PC, Krleza-Jeric K, et al. SPIRIT 2013 statement: defining standard protocol items for clinical trials. Ann Intern Med. 2013;158(3):200-7. Epub 2013/01/09. doi: 10.7326/0003-4819-158-3-201302050-00583. PubMed PMID: 23295957; PubMed Central PMCID: PMCPMC5114123.

2. Chan AW, Tetzlaff JM, Gotzsche PC, Altman DG, Mann H, Berlin JA, et al. SPIRIT 2013 explanation and elaboration: guidance for protocols of clinical trials. BMJ. 2013;346:e7586. Epub 2013/01/11. doi: 10.1136/bmj.e7586. PubMed PMID: 23303884; PubMed Central PMCID: PMCPMC3541470.
