# Supplementary material for: Nonregistration, discontinuation, and nonpublication of randomized trials: A repeated metaresearch analysis
Source: PLoS Med. 2022 Apr 27;19(4):e1003980. doi: 10.1371/journal.pmed.1003980 (PMC9094518; doi:10.1371/journal.pmed.1003980)
Supplement: S2 Table — (DOCX) [file pmed.1003980.s008.docx]

**S2 Table: Registration, completion, and publication status of randomised controlled trials approved in 2012 stratified by country of ethical approval.**

|  | **RCTs approved in Switzerland (n=165)** | **RCTs approved in the UK (n=89)** | **RCTs approved in Germany (n=37)** | **RCTs approved in Canada (n=35)** | **All included RCTs (n=326)** |
| --- | --- | --- | --- | --- | --- |
| **Registration status** |  |  |  |  |  |
| Registered | 152 (92.1%) | 85 (95.5%) | 36 (97.3%) | 34 (97.1%) | 307 (94.2%, 91.0-96.5%) |
| Prospectively registered | 138 (83.6%) | 72(80.9%) | 33 (89.2%) | 31 (88.6%) | 274 (84.0%, 79.6-87.9%) |
| Retrospectively registered | 14 (8.5%) | 13 (14.6%) | 3 (8.1%) | 4 (11.4%) | 33 (10.1%, 7.1-13.9%) |
| Not registered | 13 (7.9%) | 4 (4.5%) | 1 (2.7%) | 1 (2.9%) | 19 (5.9%, 3.5-9.0%) |
| **Completion status** |  |  |  |  |  |
| Completed | 105 (63.6%) | 58 (65.2%) | 19 (51.4%) | 21 (60.0%) | 203 (62.3%, 56.8-67.6%) |
| Discontinued | 50 (30.3%) | 25 (28.1%) | 11 (29.7%) | 12 (34.3%) | 98 (30.1%, 25.1-35.4%) |
| Unclear | 10 (6.1%) | 6 (6.7%) | 7 (18.9%) | 2 (5.7%) | 25 (7.7%, 5.0-11.1%) |
| **Results availability** |  |  |  |  |  |
| Peer reviewed publication | 133 (80.6%) | 72 (80.9%) | 24 (64.9%) | 28 (80.0%) | 256 (78.5%, 73.7-82.8%) |
| In clinical trial registry | 82 (49.7%) | 48 (53.9%) | 24 (64.9%) | 18 (51.4%) | 173 (53.1%, 47.5-58.6%) |
| As peer reviewed publication and in  clinical trial register | 74 (44.9%) | 39 (43.8%) | 17 (46.0%) | 15 (42.9%) | 145 (44.5%, 39.0-50.1%) |
| Results not available (neither as  publication nor in clinical trial register) | 26 (15.8%) | 11 (12.4%) | 7 (18.9%) | 5 (13.9%) | 42 (12.9%, 9.4-17.0%) |
| **Neither registered nor published** | 10 (6.1%) | 3 (3.4%) | 1 (2.7%) | 1 (2.9%) | 15 (4.6%, 2.6-7.5%) |
| **Not published in journal but registered** | 22/32 (68.8%) | 14/17 (82.4%) | 12/13 (92.3%) | 6/7 (85.7%) | 55/70 (78.6%, 67.1-87.5%) |
| **Not published in journal but results available in registry** | 8/32 (25.0%) | 10/18 (55.6%) | 7/13 (53.9%) | 3/7 (42.9%) | 28/70 (40.0%, 28.5-52.4%) |

Abbreviations: RCT=Randomized clinical trial
