# Supplementary material for: Nonregistration, discontinuation, and nonpublication of randomized trials: A repeated metaresearch analysis
Source: PLoS Med. 2022 Apr 27;19(4):e1003980. doi: 10.1371/journal.pmed.1003980 (PMC9094518; doi:10.1371/journal.pmed.1003980)
Supplement: S3 Table — (DOCX) [file pmed.1003980.s009.docx]

**S3 Table: Association between completion of a randomised controlled trial and making the results available.**

|  | **Completed RCTs (n=203)**^a^ | **Discontinued RCTs (n=98)**^a^ | **Odds ratio (95% Confidence interval)** | ***p-value*** |
| --- | --- | --- | --- | --- |
| Results available as a peer reviewed publication | 190 (93.6%) | 66 (67.4%) | 7.08 (3.35-15.52) | <0.001 |
| Results available in clinical trial register | 118 (58.1%) | 54 (55.1%) | 1.13 (0.67-1.89) | 0.619 |
| Results available at any source (publication or in trial registry) | 198 (97.5%) | 85 (86.7%) | 6.06 (1.94-22.24) | <0.001 |
| Not published in journal but results available in registry^b^ | 8/13 (61.5%) | 19/32 (59.4%) | 1.09 (0.25-5.26) | 0.893 |

^a^Studies with unclear discontinuation status excluded

^b^ Only a sub-sample of 44 unpublished trials considered

Abbreviations: RCT=Randomized clinical trial
