# Supplementary material for: Nonregistration, discontinuation, and nonpublication of randomized trials: A repeated metaresearch analysis
Source: PLoS Med. 2022 Apr 27;19(4):e1003980. doi: 10.1371/journal.pmed.1003980 (PMC9094518; doi:10.1371/journal.pmed.1003980)
Supplement: S4 Table — (DOCX) [file pmed.1003980.s010.docx]

**S4 Table: Non-publication and discontinuation in protocols approved by ethical committees in 2012 compared to protocols approved between 2000 to 2003 [1].**

|  | **Study-protocols approved in 2012** | | | **Study-protocols approved 2000-2003** | | |
| --- | --- | --- | --- | --- | --- | --- |
|  | **Industry-sponsored RCTs** | **Investigator-sponsored RCTs** | **All RCTs** | **Industry-sponsored RCTs** | **Investigator-sponsored RCTs** | **All RCTs** |
| **Completion status** |  |  |  |  |  |  |
| Completed | 119/179 (66.5%) | 84/147 (57.1%) | 203/326 (62.3%) | 394/551 (71.5%) | 181/343 (52.8%) | 575/894 (64.3%) |
| Discontinued | 57/179 (31.8%) | 41/147 (27.9%) | 98/326 (30.1%) | 119/551 (21.6%) | 130/343 (37.9%) | 249/894 (27.9%) |
| Unclear | 3/179 (1.7%) | 22/147 (15.0%) | 25/326 (7.7%) | 38/551 (6.9%) | 32/343 (9.3%) | 70/894 (7.8%) |
| **Results availability^a^** |  |  |  |  |  |  |
| Peer reviewed publication | 146/179 (81.6%) | 100/147 (74.8%) | 256/326 (78.5%) | 336/551 (61.0%) | 194/343 (56.6%) | 530/894 (59.3%) |
| **Reasons for discontinuation** |  |  |  |  |  |  |
| Poor recruitment^b^ | 16/57 (28%) | 20/41 (49%) | 36/98 (37%) | 40/119 (34%) | 60/130 (46%) | 100/249 (40%) |
| Futility | 15/57 (26%) | 1/41 (2%) | 16/98 (16%) | 25/119 (21%) | 12/130 (9%) | 37/249 (15%) |
| Harm | 5/57 (9%) | 1/41 (2%) | 6/98 (6%) | 17/119 (14%) | 7/130 (5%) | 24/249 (10%) |
| Organisational/strategic reasons | 6/57 (11%) | 0/41 (0%) | 6/98 (6%) | 20/119 (17%) | 16/130 (12%) | 36/249 (14%) |
| Benefit | 2/57 (4%) | 1/41 (2%) | 3/98 (3%) | 2/119 (2%) | 7/130 (5%) | 9/249 (4%) |
| External evidence | 0/57 (0%) | 3/41 (7%) | 3/98 (3%) | 6/119 (5%) | 2/130 (2%) | 8/249 (3%) |
| Limited resources | 0/57 (0%) | 1/41 (2%) | 1/98 (1%) | 1/119 (1%) | 4/130 (3%) | 5/249 (2%) |
| Unclear | 13/57 (23%) | 14/41 (34%) | 27/98 (28%) | 6/119 (5%) | 18/130 (14%) | 24/249 (10%) |
| Other | 0/57 (0%) | 0/41 (0%) | 0/98 (0%) | 2/119 (2%) | 4/130 (3%) | 6/249 (2%) |

^a^ Registries were not yet established in 2000-2003, hence results from registries are not considered in this comparison.

^b^ Two studies that stated slow recruitment as reason for discontinuation mentioned in addition another reason (i.e. organisational/strategic reasons n=1; external evidence n=1).

Abbreviations: RCT= Randomized clinical trials

**Reference:**

1. Kasenda B, von Elm E, You J, Blumle A, Tomonaga Y, Saccilotto R, et al. Prevalence, characteristics, and publication of discontinued randomized trials. JAMA. 2014;311(10):1045-51. Epub 2014/03/13. doi: 10.1001/jama.2014.1361. PubMed PMID: 24618966.
