# Supplementary material for: Nonregistration, discontinuation, and nonpublication of randomized trials: A repeated metaresearch analysis
Source: PLoS Med. 2022 Apr 27;19(4):e1003980. doi: 10.1371/journal.pmed.1003980 (PMC9094518; doi:10.1371/journal.pmed.1003980)
Supplement: S5 Table — (DOCX) [file pmed.1003980.s011.docx]

**S5 Table: Factors associated with a) publishing main results in a peer reviewed journal, b) discontinuation of trials due to poor recruitment, and c) discontinuation of trials due to preventable reasons. UK samples excluded because variable “CTU/CRO support” was not assessed.**

| **Characteristics** |  |  | **Univariable** | | | **Multivariable** | | |
| --- | --- | --- | --- | --- | --- | --- | --- | --- |
|  |  |  | **OR** | **95% CI** | ***P-value*** | **OR** | **95% CI** | ***P-value*** |
| **Non-publication in a peer reviewed journal** | **RCT not published in peer reviewed journal (n=52)** | **RCTs published in a peer reviewed journal (n=185)** |  |  |  |  |  |  |
| Proportion of adequate SPIRIT reporting, median (IQR)^a^ | 0.69 (0.52, 0.75) | 0.73 (0.65, 0.79) | 0.64 | 0.51-0.81 | <0.001 | 0.67 | 0.49-0.92 | 0.014 |
| Planned target sample size, median (IQR)^b^ | 155 (63, 294) | 375 (120, 800) | 0.99 | 0.97-1.01 | 0.249 | 0.99 | 0.98-1.01 | 0.421 |
| CTU/CRO support | 24/52 (46.2%) | 112/185 (60.5%) | 0.56 | 0.30-1.04 | 0.066 | 0.87 | 0.42-1.78 | 0.698 |
| Placebo controlled (vs. not placebo controlled) | 23/52 (44.2%) | 77/185 (41.6%) | 1.11 | 0.60-2.07 | 0.736 | 1.68 | 0.82-3.42 | 0.153 |
| Single-centre (vs. multicentre) | 15/52 (28.9%) | 26/185 (14.1%) | 2.48 | 1.20-5.14 | 0.015 | 1.23 | 0.48-3.15 | 0.671 |
| Reported recruitment projection | 14/52 (26.9%) | 65/185 (35.1%) | 0.68 | 0.34-1.35 | 0.269 | 0.91 | 0.43-1.95 | 0.812 |
| Industry-sponsored | 23/52 (44.2%) | 111/185 (60.0%) | 0.53 | 0.28-0.98 | 0.044 | 0.89 | 0.38-2.10 | 0.171 |
| **Discontinued due to poor recruitment** | **RCTs discontinued due to poor recruitment (n=29)** | **RCTs not discontinued due to poor recruitment (n=189)^c^** |  |  |  |  |  |  |
| Proportion of adequate SPIRIT reporting, median (IQR)^a^ | 0.67 (0.60, 0.78) | 0.73 (0.65, 0.79) | 0.78 | 0.56-1.08 | 0.130 | 0.93 | 0.59-1.45 | 0.740 |
| Planned target sample size, median (IQR)^b^ | 140 (78, 400) | 360 (124, 800) | 0.93 | 0.85-1.02 | 0.117 | 0.94 | 0.87-1.01 | 0.107 |
| CTU/CRO support | 18/29 (62.1%) | 110/189 (58.2%) | 1.18 | 0.53-2.63 | 0.694 | 1.97 | 0.75-5.16 | 0.168 |
| Placebo controlled (vs. not placebo controlled) | 12/29 (41.4%) | 83/189 (43.9%) | 0.90 | 0.41-1.99 | 0.798 | 1.37 | 0.57-3.34 | 0.482 |
| Single-centre (vs. multicentre) | 7/29 (24.1%) | 25/189 (13.2%) | 2.09 | 0.81-5.39 | 0.129 | 0.84 | 0.26-2.71 | 0.774 |
| Reported recruitment projection | 9/29 (31.0%) | 60/189 (31.8%) | 0.97 | 0.42-2.25 | 0.939 | 0.96 | 0.37-2.50 | 0.932 |
| Industry-sponsored | 11/29 (37.9%) | 121/189 (64.0%) | 0.34 | 0.15-0.77 | 0.009 | 0.28 | 0.10-0.83 | 0.021 |
| **Discontinued due to preventable reasons** | **RCTs discontinued due to preventable reason (n=53)**^d^ | **RCTs not discontinued due to preventable reason (n=165)^c,d^** |  |  |  |  |  |  |
| Proportion of adequate SPIRIT reporting, median (IQR)^a^ | 0.69 (0.61, 0.78) | 0.74 (0.65, 0.79) | 0.75 | 0.58-0.99 | 0.042 | 0.90 | 0.63-1.29 | 0.567 |
| Planned target sample size, median (IQR)^b^ | 200 (90, 560) | 360 (124, 800) | 0.99 | 0.98-1.01 | 0.381 | 1.00 | 0.98-1.01 | 0.531 |
| CTU/CRO support | 29/53 (54.7%) | 99/165 (60.0%) | 0.81 | 0.43-1.50 | 0.497 | 1.04 | 0.51-2.13 | 0.914 |
| Placebo controlled (vs. not placebo controlled) | 23/53 (43.4%) | 72/165 (43.6%) | 0.99 | 0.53-1.85 | 0.976 | 1.28 | 0.64-2.56 | 0.479 |
| Single-centre (vs. multicentre) | 11/53 (20.8%) | 21/165 (12.7%) | 1.80 | 0.80-4.02 | 0.155 | 0.91 | 0.34-2.45 | 0.847 |
| Reported recruitment projection | 13/53 (24.5%) | 56/165 (33.9%) | 0.63 | 0.31-1.28 | 0.202 | 0.63 | 0.28-1.36 | 0.237 |
| Industry-sponsored | 25/53 (47.2%) | 107/165 (64.9%) | 0.48 | 0.26-0.91 | 0.023 | 0.47 | 0.20-1.09 | 0.079 |

^a^In increments of 10%

^b^In increments of 100

^c^Studies with unclear discontinuation status excluded

^d^ Counting the following reasons as not preventable: Futility, Harm, Benefit, External evidence. Counting the following as preventable: Poor recruitment, organisational/strategic reasons, limited resources, and unclear reasons (assuming that discontinuation due to unclear reasons was mainly due to non-data driven reasons [1]).

Abbreviations: RCT= Randomized clinical trials; OR= Odds ratio; CI= Confidence interval; IQR= Interquartile range; SPIRIT= Standard Protocol Items: Recommendations for Interventional Trials[2, 3]; CTU= Clinical trial unit; CRO= Contract Research Organization

References:

1. Briel M, Olu KK, von Elm E, Kasenda B, Alturki R, Agarwal A, et al. A systematic review of discontinued trials suggested that most reasons for recruitment failure were preventable. J Clin Epidemiol. 2016;80:8-15. Epub 2016/08/09. doi: 10.1016/j.jclinepi.2016.07.016. PubMed PMID: 27498376.

2. Chan AW, Tetzlaff JM, Gotzsche PC, Altman DG, Mann H, Berlin JA, et al. SPIRIT 2013 explanation and elaboration: guidance for protocols of clinical trials. BMJ. 2013;346:e7586. Epub 2013/01/11. doi: 10.1136/bmj.e7586. PubMed PMID: 23303884; PubMed Central PMCID: PMCPMC3541470.

3. Chan AW, Tetzlaff JM, Altman DG, Laupacis A, Gotzsche PC, Krleza-Jeric K, et al. SPIRIT 2013 statement: defining standard protocol items for clinical trials. Ann Intern Med. 2013;158(3):200-7. Epub 2013/01/09. doi: 10.7326/0003-4819-158-3-201302050-00583. PubMed PMID: 23295957; PubMed Central PMCID: PMCPMC5114123.
