# Supplementary material for: Nonregistration, discontinuation, and nonpublication of randomized trials: A repeated metaresearch analysis
Source: PLoS Med. 2022 Apr 27;19(4):e1003980. doi: 10.1371/journal.pmed.1003980 (PMC9094518; doi:10.1371/journal.pmed.1003980)
Supplement: Attachment — Submitted filename: speich.pdf [file pmed.1003980.s012.pdf]

This paper describes the association between clinical trials approved in four countries and various outcomes.

I was asked for a statistical report and I interpret that to include all aspects of the design and conduct of the study.

1 Points of detail

Page 6 I find it odd to say that non–publication has improved. Perhaps 'non–publication of RCTs has declined [...]'?

Page 7 Interestingly this study does not seem to have been registered in, for instance, PROSPERO. Of course this does not matter since the protocol was published which fulfils the same purpose but have the authors considered whether any of the unregistered trials had a published protocol?

Page 10 I think it would be helpful to state why the UK was excluded from one of the analyses.

Page 14 The authors' bibliographical management software seems to have mangled reference 5.

Page 20, Table 4 Are the footnotes correct? Is proportion of adequate SPIRIT really per 100 and Planned target sample size per 10%?

Table S1 This reveals some differences between countries so it might be worthwhile to consider including country as a covariate in the models.

I must say I was surprised by the high proportion of discontinued trials which were published in some form. Authors and editors get a bad press for only publishing things which achieve some arbitrary level of statistical significance so the fact that two–thirds appear is reassuring.

2 Points of more substance

2.1 Is discontinuation necessarily a bad thing?

The authors clearly think so as in the abstract (page 6) they state:

> Premature trial discontinuation has not improved in the past decade.

I think the picture is more nuanced and I take each reason separately below. I assume without further argument that we all agree that before a trial the

team should be in equipoise and that we should be aiming for a net benefit both for patients in the trial and for subsequent patients with the same condition.

2.1.1 Not preventable reasons

Futility I would argue that stopping early for futility when it is clearly the case is a good thing. It is inevitable that some trials will find no perceptible difference between the conditions because before the trial started we were in equipoise. Stopping the trial and reporting that fact saves prospective patients in the trial from undergoing potentially onerous extra visits and investigations for no benefit to them or anyone else.

Harm The arguments here are similar to those for futility but rather stronger since here we know that continuation is going to harm more people.

Benefit This is almost the converse of stopping for harm as if the trial continues then people in the control group are going to fail to benefit and hence suffer harm relative to the treatment group.

External evidence Not much to say here.

2.1.2 Preventable reasons

Poor recruitment It is hard to know without more detail how preventable this was. I assume the authors have in mind something like the cautionary tale of Blackwell and Shepherd (1967) where 518 people were admitted and thought to be eligible but only 8 could be recruited. This was a long while ago and one would hope we could do better now. But there are situation like those outlined in the systematic review of Grant et al. (1996) where all six trials stopped early for poor recruitment, a fact which it would have been hard to predict in advance since they occurred mostly simultaneously. Trying to pilot this is also tricky since there is a gap between asking people 'Would you agree to go into a trial if the hypothetical situation arose?' versus asking them 'Will you go into the trial right now?'.

Organizational/strategic Not clear what exactly this covers but health service reorganisations occur with some rapidity in the UK and can make a trial unviable. Issues like the hospital replacing the MRI scanner with an incompatible version presumably fall under this category so I think we need more detail before coming to a final judgment about whether they are preventable.

Limited resources This must depend on why the resources were inadequate.

Unclear reasons No comment

2.2 Disclaimer

I feel in fairness I should state that I have been involved in several Data Monitoring and Ethics Committees and we have had to take the difficult decision for one of them to recommend discontinuation for harm (DiPALS Writing Committee, on behalf of the DiPALS Study Group Collaborators, 2015). I have also been involved in one where the issue of futility is arising but I cannot give details for confidentiality reasons. I hope I have not allowed my personal experiences to influence me too much.

3 Summary

I do not have major concerns about the statistical analysis and putting this sort of information in the public domain is clearly a good thing. I do feel that unless the authors can provide more detail about exactly why each trial was discontinued and confirm which were genuinely a bad thing the language needs to be more neutral.

Michael Dewey

References

B Blackwell and M Shepherd. Early evaluation of psychotropic drugs in man: a trial that failed. *Lancet*, 290:819–822, 1967.

DiPALS Writing Committee, on behalf of the DiPALS Study Group Collaborators. Safety and efficacy of diaphragm pacing in patients with respiratory insufficiency due to amyotrophic lateral sclerosis (DiPALS): a multicentre, open–label, randomised controlled trial. *Lancet Neurology*, 14:883–892, 2015.

A Grant, Z J Penn, and P J Steer. Elective or selective caesarian delivery of the small baby? A systematic review of the controlled trials. *British Journal of Obstetrics and Gynaecology*, 103:1197–1200, 1996.
