# Supplementary material for: Nonregistration, discontinuation, and nonpublication of randomized trials: A repeated metaresearch analysis
Source: PLoS Med. 2022 Apr 27;19(4):e1003980. doi: 10.1371/journal.pmed.1003980 (PMC9094518; doi:10.1371/journal.pmed.1003980)
Supplement: Attachment — Submitted filename: p-b-p response 2022-02-23.docx [file pmed.1003980.s013.docx]

Dear Dr. Davidson,

Dear Editors,

Dear Peer-reviewers,

We thank you for carefully assessing our submitted study and for considering our manuscript for potential publication in *PLOS Medicine*. We have implemented and clarified the helpful comments to further strengthen our manuscript. Please see our responses below in bold (blue color). Of note, all line numbers are referring to the track-changed version of our manuscript.

Comments from the academic editor:

1) I would suggest that the authors (1) better describe the sample so as to help understand if it is generalizable and (2) dig into the 'research waste' idea a bit more, since a major limitation of the study is any details on the RCTs themselves to understand how many patients accrued, what the flaws in the study were, etc. If no patients accrued, then where is the 'waste'?

**Response**: We have now described the sample in more detail. In short, we have now listed all participating ethical committees, and we have explained that for most ethics committees we included all eligible protocols approved in 2012 in our study and with three ethical committees we drew a random sample of eligible protocols for feasibility reasons. Furthermore, we added more details on the interventions and different medical fields of included RCTs in Table 1, presenting trial characteristics. In addition, we highlight explain now the ASPIRE project from which the sample is drawn in more detail (see revised manuscript, Table 1, and lines 226-229, and 217-223). We describe now also that only RCTs that started patient enrollment were included in our study (see revised manuscript, lines 234-236). In addition, we have expanded the paragraph about research waste in the introduction section (see revised manuscript, lines 201-205). With respect to the editor’s question about research waste in trials without any accrued patients, we think that the planning of a trial with writing of the protocol and the review process by an ethical committee can already consume considerable resources, which are eventually wasted. In a previous study of ours (Speich et al. *Resource use, costs, and approval times for planning and preparing a randomized clinical trial before and after the implementation of the new Swiss human research legislation,* PLoS one 2019 [1]) we empirically estimated RCT preparation costs of USD 72’0000.

Requests from the editors:

2) Please report your SR/MA according to the PRISMA guidelines provided at the EQUATOR site.

<http://www.equator-network.org/reporting-guidelines/prisma/>

Please provide the completed PRISMA checklist. I appreciate that the nature of the study means some items may be non-applicable.

**Response**: As our study is a meta-research study and not a systematic review assessing the effect of a medical intervention on health outcomes, not all PRISMA-items are applicable. As requested by the editors, we have added a PRISMA checklist reporting all applicable reporting items.

3) When completing the checklist, please use section and paragraph numbers, rather than page numbers.

**Response**: We have filled out the reporting checklist, using section and paragraph numbers as requested.

4) Please add the following statement, or similar, to the Methods: "This study is reported as per the Preferred Reporting Items for Systematic Reviews and Meta-Analyses (PRISMA) guideline (S1 Checklist)."

**Response**: We have included the statement in our revised manuscript (see revised manuscript, lines 302-305).

5) If possible, please update your search end date from March 2021 to the present time.

**Response**: We followed the suggestion from the editors and have updated our search in February 2022 (see revised manuscript, lines 131-133 and 258-259). We have therefore redone all analyses. The overall interpretation of the results remains unchanged. Please note that with the update one of the 327 RCTs was excluded from our sample, as we found new evidence indicating that one study was never started.

6) The Data Availability Statement (DAS) requires revision. Please also include an appropriate contact (web or email address) for inquiries (this cannot be a study author).

**Response**: Thank you for the opportunity to better clarify this point. We conducted this study in close collaboration with research ethics committees and have written agreements with them. Therefore, we revised the DAS as follows:

*“All participating ethics committees were project partners and granted us access to confidential study protocols under the condition that only aggregated data will be made publicly available. All investigators signed a corresponding confidentiality agreement with ethics committees. All aggregated data that were used for this study are presented in Tables and Figures of the manuscript or the appendix. The appendix also includes the used statistical code (STATA; Appendix 4).”* (see revised manuscript, lines 472-477).

In case the editors have any suggestions how we can better resolve this issue, we are happy to consider these.

7) Abstract Methods and Findings:

* Please include the study design.

* Please include the key databases searched.

* Please include relevant p-values when quantifying your results.

* In the last sentence of the Abstract Methods and Findings section, please describe the main limitation(s) of the study's methodology.

**Response**: We have now included the study design (i.e. meta-research study; revised manuscript line 129-130). As requested by the editors, we have also included all searched databases and added a sentence highlighting the main limitation (see revised manuscript, lines 133-137 and 147-148). However, as several databases were searched this has increased the word-count of the abstract considerably. In addition, as requested we have added missing p-values (see revised manuscript, lines 150-152).

8) Abstract Conclusions:

* Please address the study implications without overreaching what can be concluded from the data; the phrase "In this study, we observed ..." may be useful.

**Response**: Following the advice from the editors, we have rephrased the conclusions carefully (“*We have observed that*…”; see revised manuscript, lines 155-160).

9) Please remove the Funding and Competing Interests sections from the main text, this information is captured as metadata from the submission form.

**Response**: We have removed the Funding and Competing Interests section from the main text (see revised manuscript, lines 106-120). We have marked in the SPIRIT-checklist that this information was submitted as meta-data to the journal.

10) At this stage, we ask that you include a short, non-technical Author Summary of your research to make findings accessible to a wide audience that includes both scientists and non-scientists. The Author Summary should immediately follow the Abstract in your revised manuscript. This text is subject to editorial change and should be distinct from the scientific abstract. Please see our author guidelines for more information: <https://journals.plos.org/plosmedicine/s/revising-your-manuscript#loc-author-summary>

**Response**: Thank you, we have added an Author summary as requested (see revised manuscript, lines 162-187).

11) Please define the abbreviations in Figure 1.

**Response**: Apologies for missing to define the abbreviations. We have included them now in the legend of Figure 1 in the revised manuscript (see line 606).

12) Please include the relevant p values for the results presented on lines 262 and 267.

**Response**: We have added the missing p-values (see revised manuscript, lines 338-340 and 349-351).

13) Line 296: Please do not present new data in the Discussion, relocate this to the Results.

**Response**: Apologies for introducing data in the discussion (i.e. comparison to the study on RCTs approved in 2000-2003). We have now, as suggested, relocated this comparison to the results section (see revised manuscript, lines 342-345).

14) Please expand your Discussion to include implications and next steps for research, clinical practice, and/or public policy (this should follow your Strengths and Limitations section and precede your Conclusions).

**Response**: We have now added a paragraph called “Implications” within the discussion (see revised manuscript, line 427-445).

15) Please relocate the Patient and Public Involvement section to the Methods.

**Response**: We have now relocated the Patient and public involvement section to the Methods (see revised manuscript, lines 302-304).

16) Reference #5: Please remove the COI information from this reference.

**Response**: Thank you for noting this; we have now removed the unnecessary reference information (see revised manuscript, lines 512-515; of note: no track change markings due to endnote).

17) Supplementary materials: Please update your supplementary references to follow our reference guidelines (<https://journals.plos.org/plosmedicine/s/submission-guidelines#loc-references>)

**Response**: The references in the updated appendix are now adhering to the PLOS-style (see revised appendix).

**Comments from the reviewers:**

**Reviewer #1** I thank the editor to having the opportunity to review this manuscript.

The authors report the fate (with regards to completion, quality, and publication) of 327 randomized controlled trials (RCTs) approved by research ethics committees in 4 countries in 2012. The assessment was conducted in 2021.

This work is of high interest since there are very few studies that have access to the protocols approved by RECs ─most similar analysis only have access to the published (on articles or posted on registries) results. The results reported are also of interest since the same authors conducted a similar analysis with RCTs approved in 2003-2004 allowing to compare the outcome of both cohorts. The manuscript is very easy to follow, well written and focused. I would stress the highly useful data reported regarding reasons for discontinuation, which is hardly found in other reports.

This manuscript should affect public health policy with regards to the low fulfilment of posting trial results of investigator-initiated trials on trial registries. RECs should be involved on this.

**Response**: We thank reviewer 1 for the careful review of our manuscript, the positive feedback, and the helpful comments, which we addressed below.

1) I have no major concerns on how the data was presented and analyzed, except for the following: In Acknowledgement, one can read the RECs involved. However, it is not clear how the UK NHSHRA provided the 89 RCTs included in this analysis. I would bet that there were many more than 89 RCTs approved in the UK in 2012; then, which were the criteria to choose those 89 RCTs? This is a very relevant information that readers should know and that should be included in Methods.

**Response**: We agree that this point was not clear and have now specified in the acknowledgment section that UK RCTs were collected from the Bristol office of the UK National Research Ethics Service (see revised manuscript, lines 226-229). In the Methods section we have now described the sample in more detail (see revised manuscript, lines 217-223 and 231-236) and additionally refer to our published study protocol [2].

2) In addition, I would like to stress that mentioning that investigator-initiated trials usually fail on having trial results posted on the registries should be included in both the abstract and conclusion. This should be one of the main messages of this report.

**Response**: We agree that this result should be presented more prominently. We have now included this point in the abstract and the conclusions of the revised manuscript as suggested by the reviewer (see revised manuscript, lines 147-148). As indicated by the editors we have now added a section within the discussion, mentioning implications and next steps where we underline this result as well as indicating what should change (see revised manuscript, lines 433-439). In addition, we also underline this point in the Authors Summary which we have added to the manuscript (see revised manuscript, lines 178-179 and 186-187).

Below I mentioned a few suggestions that, I believe, would be of interest to many readers.

Minor comments:

3) Seems to me that this research includes RCTs on medicines only (Industry-sponsored may also include trials on devices, but I believe that the research was conducted on RCTs assessing medicines only). That the research was conducted on medicines RCTs must be stated in the title, and in other appropriate sections of the manuscript.

**Response**: Thank you for the opportunity to clarify this point. We did not only include RCTs with drug interventions; in fact, we included randomized clinical trials that assessed all kinds of interventions to evaluate their effects on health outcomes. We stated this in our manuscript as follows: “*We included RCTs in which participants were randomly assigned to different interventions (or an intervention and control group) to evaluate their effect on health outcomes.*” (see revised manuscript, lines 229-231) and in more detail in the referenced study protocol. To underline this point, we have now also included the type of intervention in Table 1 presenting trial characteristics (see revised manuscript Table 1 and line 312-314).

Abstract

4) The abstract should report the number (and percentage) of RCTs by trial phase (2, 3 or 4) and the number (and percentage) of RCTs that were not published but of which the results are posted on the registries, and on RCTs that were published and posted the results on the registries. (This latter is relevant, since many RCTs could be published in subscription-only journals, so access to the results is limited to a minority of interested parties).

Reporting the clinical development phases (2, 3 and 4) of the RCTs assessed is relevant, since the clinical usefulness of phase 3 and 4 RCTs is much higher than of phase 2 trials.

**Response**: Even though the clinical usefulness of phase 3 and 4 trials may be higher than of phase 2 trials, we believe that with respect to non-registration and non-publication they both matter. Independent of the study phase, not registering a study and not publishing study results is considered malpractice, is unethical and contributes to research waste (see revised manuscript, line 201-205). In general, we agree with the reviewer that it would be interesting to see the results stratified by trial phases. However, as clarified in our response to your comment 3 above we included all kinds of trial interventions (not only drug trials), so the phases do often not apply. In addition, even for drug trials we realized that the trial phase was often not reported in the trial protocol or that the labelling was not accurate. Therefore, we decided that reporting trial phases for included RCTs would not be sensible.

Introduction

5) In addition to the ICJME requirement of trial registration, authors should mention that both the EU and North American regulations (Canada, USA) requested registration of trials on medicines. I do not know whether the Swiss regulation requested registration as of 2012, but is likely it did.

**Response**: Thank you for raising this point. We have now added a sentence in the introduction clarifying that trial registration is requested by law in many parts of the world (see revised manuscript, lines 195-197). In the results section we highlight that the proportion of unregistered trials was highest in Switzerland (see revised manuscript, lines 345-348), and in the discussion section we put this result in context by stating that the registration in Switzerland is only mandatory since 2014 (see revised manuscript, lines 422-425).

Methods

6) Authors should report the number of research ethics committees by country involved in this research.

**Response**: We have now listed all research ethics committees for each country (see revised manuscript, lines 226-229).

7) Lines 210-211.- Authors should inform who, among the authors, were the 'pairs of reviewers'.

**Response**: We apologize for our imprecise wording. All searches and data extractions were conducted in duplicate. However, there were no fixed “pairs of reviewers”. We have, re-phrased both sentences that mentioned “pairs of reviewers” (see revised manuscript, lines 238-240 and 269-271).

8) Among those RCTs that were registered in more than one registry, how many posted the results in all registries?

**Response**: We agree with the reviewer that this is an interesting question. We further wondered, how consistent the registered information was across registries for RCTs that were registered in more than one registry. We, therefore, conducted a separate study on RCTs registered in several registries, which was recently published under the title: “*Reliability of Trial Information Across Registries for Trials With Multiple Registrations: A Systematic Review*” [3].

Analysis

9) (General comment: Since authors had complete data for all RCTs, I do believe it is inappropriate to present confidence intervals as if these were point estimates randomly sampled from a larger population. Do authors agree with this?)

**Response**: In the revised manuscript we have now explained in more detail how the sample was selected. In short, we included all eligible RCT protocols from the ethics committees in Basel, Bellinzona, Bern, Geneva, Lausanne, St. Gallen, and Thurgau (Switzerland) and the Bristol office of the UK National Research Ethics Service responsible for 19 research ethics committees (UK). From Freiburg (Germany), Hamilton (Canada) and Zürich (Switzerland) a random sample of protocols was selected (see revised manuscript, lines 226-233). In addition, given the fact that we chose only one year (2012) for approved RCTs to be included in our study, and that not all ethical committees were included in the selected countries (except Switzerland), we think it makes sense to present confidence intervals, reflecting the possible variance of our data.

10) Line 221.- Are the pre-specified independent variables in their models based on the previous analysis by the same authors or by published reports of other investigators?

**Response**: We agree that this was not clearly described in the previous version of our manuscript. We have now clarified that the variable “proportion of SPIRIT items reported” was pre-specified in the study protocol and that the other variables were selected following our previous analyses on study protocols that were approved between 2000-2003 (see revised manuscript, lines 283-291).

11) Lines 225-228.- What was the reason supporting the inclusion of a post-hoc analysis? That this analysis was done excluding UK RCTs (as reported in line 264) should be included here. I wonder how relevant this post-hoc analysis is.

**Response**: Our hypothesis was that trials that received support from a CTU or CRO might have performed better in terms of discontinuation or non-publication. Since for the UK sample this information about CTU/CRO support was not provided, all UK RCTs were excluded from this analysis. We did not find any evidence that CTU or CRO support changed the non-publication or discontinuation rate. We would prefer keeping these results in the manuscript at that current stage. We have now rephrased the Analysis section in which we explain the rationale for choosing the variables and why we excluded the UK sample for this specific analysis. (see revised manuscript, line 283-297).

12) Line 230.- p>0.05 should be p<0.05 (?)

**Response**: We thank the reviewer for spotting this error (see revised manuscript, line 299).

Results

13) Lines 240-241.- The number of RCTs approved by REC and clinical development phases (2, 3 or 4) should be included in Table 1. Although of less relevance, I would also suggest reporting whether the trials were conducted in hospitals and/or primary care centers ─ this info might be difficult to know in some trials.

**Response**: With respect to trial phase, please see our response to your comment 4 above. We did not extract information if the trials were conducted in hospitals or primary care centers. To give the readers more information about the type of trials included, we have added the type of intervention and the medical field in Table 1 (see revised manuscript Table 1 and line 312-314).

14) Line 250.- 4 published trials were not registered. It would be of interest to know whether the journals, at the time of publication, were followers of the ICMJE requirements or not as per the info included in their websites.

**Response**: We have checked the current guidelines of the four journals in which these trials are published. While three journal-guidelines do not mention that a registration is mandatory, the current guideline of one journal mentions that only registered trials will be published. We do not know if this guideline was already in place when the study was published (in 2018) since no history is available when journal guidelines were changed.

15) Line 264.- 'A separate analysis...' should read as follows: 'A separate post-hoc analysis…'

**Response**: We have now described in more detail in the analyses section how the conducted analyses were chosen (i.e. “*Variables 2-7 were selected post-hoc following our previous conducted analysis in trial protocols approved between 2000-2003 to allow for a meaningful comparison*” (see revised manuscript, lines 289-291).

16) Lines 267-268: I would suggest deleting 'compared to discontinued trials' that is mentioned at the beginning of the sentence (line 266)

**Response**: Thank you for noticing this, we have re-worded the sentence as suggested (see revised manuscript, lines 338-340 and 335-357).

Discussion

17) Line 279.- I do believe that posting the results on the registry is even more relevant than publishing them (see above). Hence, I strongly suggest mentioning that industry-sponsored trials tend to perform better in this regard as well. Maybe the emphasis should be on the bad performance of investigator-sponsored trials, rather than in the better performance of industry-sponsored trials.

**Response**: We agree and have therefore added one sentence, highlighting that “Only 1 in 6 Investigator-sponsored trials made results available in a trial registry.” (see revised manuscript, lines 363-364).

18) Lines 319-321.- I do not see the need to add this 4th limitation…since only 6% of RCTs were not registered (!). In addition, mentioning it could mislead readers as, I do believe, that in 2012 all 4 countries where the RCTs were conducted were requesting prospective registration of trials ─as well as the ICMJE requirements, mentioned in the Introduction.

**Response**: We clarified in this sentence that registration by law in Switzerland is only requested since 2014 (see revised manuscript, lines 422-425). Due to this reason, we believe that it is sensible to keep this limitation in the manuscript.

19) Line 326.- One or two of the many references that support the statement 'e.g., changing the primary outcome' should be included here.

**Response**: We have now added a reference to support this statement (i.e. Goldacre et al., 2019, *Trials* 2019; see revised manuscript, lines 448-450).

20) Line 326.- The statement '1 in 5 unpublished trials were not registered' has not enough relevance to be mentioned here (conclusions), since 94% of trials were registered (!).

**Response**: We agree that the overall proportion of 94% registered trials is good. However, trial registries should also be able to identify trials that were not published. Hence, we believe it is highly relevant that 1 out of 5 unpublished trials cannot be identified in a trial registry. Therefore, we would prefer keeping the statement as is.

21) Table 2.- In the EU, to have a RCT authorized by the regulatory agency of the country involved, an EudraCT number must have been given before study start. Hence, I would expect that the 20 RCTs that were not registered and those 34 that were retrospectively registered should have been approved by non-EU RECs. Am I correct? I do believe that the country (ies) in which these 54 RCTs were approved should be included as a footnote. This is a relevant piece of information.

**Response**: This information (i.e. number of unregistered and retrospective registered trials listed by country) is available in Appendix Table 2. While retrospective registrations seemed to be equally distributed amongst countries, we found that Switzerland had a higher proportion of unregistered trials (see our response to your comment 5). We have now added a statement within the manuscript to highlight these results “*Switzerland had more unregistered trials (7.9%; 13/165), compared to the other countries (UK 4.5%, 4/89; Germany 2.7%, 1/37; Canada 2.9%, 1/35); otherwise results were comparable in terms of discontinuation and non-publication amongst the 4 included countries (Appendix Table 2).*” (see revised manuscript, lines 345-348).

22) Table 3.- An additional column reporting 'results available in a peer reviewed publication + clinical trial register' would be of great interest. Although regulations request to post clinical trial results on the registries, it is well known ─and the results of this research show─ that many sponsors publish the results in articles but forget to post them on the registry.

**Response**: The information “Results available as peer reviewed publication and in clinical trial register” are presented in Table 2. Table 3 gives a more detailed overview of trials that were prematurely discontinued. As suggested by the reviewer, we have added now a column in Table 3 which gives information on how many discontinued RCTs were published as a peer-reviewed publication and results posted in a clinical trial registry (see revised manuscript, Table 3).

**Reviewer #2** (Michael Dewey)**:**

This paper describes the association between clinical trials approved in four countries and various outcomes. I was asked for a statistical report and I interpret that to include all aspects of the design and conduct of the study.

**Response**: We thank reviewer 2 for the careful evaluation of our manuscript.

1 Points of detail
1) Page 6 I find it odd to say that non–publication has improved. Perhaps ‘non–publication of RCTs has declined [...]’?

**Response**: We have changed the wording as suggested and state that “*Non-publication of RCTs has declined,…*” (see revised manuscript, lines 156-157).

2) Page 7 Interestingly this study does not seem to have been registered in, for instance, PROSPERO. Of course this does not matter since the protocol was published which fulfils the same purpose but have the authors considered whether any of the unregistered trials had a published protocol?

**Response**: We decided to publish the study protocol especially as it was not possible to register this meta-research study in PROSPERO. PROSPERO accepts only systematic reviews for registration that assess the effect of an intervention on a health outcome. We have also assessed in a separate sub-study, how many protocols were made publicly available at what time point they were made available. This sub-study entitled “*The availability of randomized controlled trial protocols and the timing of their accessibility: – A meta-research study*” is currently under review at the Journal of Clinical Epidemiology. From the 70 unpublished trials 9 made their protocol publicly available.

3) Page 10 I think it would be helpful to state why the UK was excluded from one of the analyses.

**Response**: We excluded the UK protocols only in the analysis in which we assessed if support from a Clinical Trial Unit (CTU) or a Contract Research Organization (CRO) (vs. no support) was associated with lower discontinuation or publication rates. We clarify now that the UK sample was excluded as we were not able to extract this information from study protocols (see revised manuscript, lines 295-297).

4) Page 14 The authors’ bibliographical management software seems to have mangled reference 5.

**Response**: Apologies for that. We have now adapted the Al-Durra et al. reference accordingly (see revised manuscript, lines 512-515).

5) Page 20, Table 4 Are the footnotes correct? Is proportion of adequate SPIRIT really per 100 and Planned target sample size per 10%?

**Response**: Reviewer 2 is absolutely correct that this should be the other way around. Thank you for noticing. We have corrected the footnote accordingly (see revised manuscript, line 636-637).

6) Table S1 This reveals some differences between countries so it might be worthwhile to consider including country as a covariate in the models.

**Response**: The separate characteristics that are listed in Table S1 for each country are all considered as covariates in our models (with the exception of study design which appears comparable across countries). Hence, we would prefer not to add more variables to our model.

7) I must say I was surprised by the high proportion of discontinued trials which were published in some form. Authors and editors get a bad press for only publishing things which achieve some arbitrary level of statistical significance so the fact that two–thirds appear is reassuring.

Points of more substance

Is discontinuation necessarily a bad thing?

The authors clearly think so as in the abstract (page 6) they state:

Premature trial discontinuation has not improved in the past decade.

I think the picture is more nuanced and I take each reason separately below. I assume without further argument that we all agree that before a trial the team should be in equipoise and that we should be aiming for a net benefit both for patients in the trial and for subsequent patients with the same condition.

Not preventable reasons

Futility I would argue that stopping early for futility when it is clearly the case is a good thing. It is inevitable that some trials will find no perceptible difference between the conditions because before the trial started we were in equipoise. Stopping the trial and reporting that fact saves prospective patients in the trial from undergoing potentially onerous extra visits and investigations for no benefit to them or anyone else.

Harm The arguments here are similar to those for futility but rather stronger since here we know that continuation is going to harm more people.

Benefit This is almost the converse of stopping for harm as if the trial continues then people in the control group are going to fail to benefit and hence suffer harm relative to the treatment group.

External evidence	Not much to say here.

Preventable reasons

Poor recruitment It is hard to know without more detail how preventable this was. I assume the authors have in mind something like the cautionary tale of Blackwell and Shepherd (1967) where 518 people were admitted and thought to be eligible but only 8 could be recruited. This was a long while ago and one would hope we could do better now. But there are situation like those outlined in the systematic review of Grant et al. (1996) where all six trials stopped early for poor recruitment, a fact which it would have been hard to predict in advance since they occurred mostly simultaneously. Trying to pilot this is also tricky since there is a gap between asking people ‘Would you agree to go into a trial if the hypothetical situation arose?’ versus asking them ‘Will you go into the trial right now?’.

Organizational/strategic Not clear what exactly this covers but health service reorganisations occur with some rapidity in the UK and can make a trial unviable. Issues like the hospital replacing the MRI scanner with an incompatible version presumably fall under this category so I think we need more detail before coming to a final judgment about whether they are preventable.

Limited resources This must depend on why the resources were inadequate.

Unclear reasons	No comment

Disclaimer

I feel in fairness I should state that I have been involved in several Data Monitoring and Ethics Committees and we have had to take the difficult decision for one of them to recommend discontinuation for harm (DiPALS Writing Committee, on behalf of the DiPALS Study Group Collaborators, 2015). I have also been involved in one where the issue of futility is arising but I cannot give details for confidentiality reasons. I hope I have not allowed my personal experiences to influence me too much.

Summary

I do not have major concerns about the statistical analysis and putting this sort of information in the public domain is clearly a good thing. I do feel that unless the authors can provide more detail about exactly why each trial was discontinued and confirm which were genuinely a bad thing the language needs to be more neutral.

**Response**: Thank you for this detailed comment. Overall we agree with the reviewer that trial discontinuation is not necessarily always a bad thing (see revised manuscript, line 280-283). Therefore, we now use a more neutral language regarding discontinuation throughout the revised manuscript.

References

B Blackwell and M Shepherd. Early evaluation of psychotropic drugs in man: a trial that failed. Lancet, 290:819–822, 1967.

DiPALS Writing Committee, on behalf of the DiPALS Study Group Collaborators. Safety and efficacy of diaphragm pacing in patients with respiratory insufficiency due to amyotrophic lateral sclerosis (DiPALS): a multicentre, open–label, randomised controlled trial. Lancet Neurology, 14:883–892, 2015.

A Grant, Z J Penn, and P J Steer. Elective or selective caesarian delivery of the small baby? A systematic review of the controlled trials. British Journal of Obstetrics and Gynaecology, 103:1197–1200, 1996.

**Reviewer #3:**

Thank you for the invitation to review this rigorous and interesting research article. In this manuscript, Speich et al. focus on an important issue - rates of completion and publication of clinical trials. As the authors note, trial discontinuation and non-publication contribute to substantial research waste. Over the past two decades, clinical trial registries have been implemented to improve transparency and journal policies related to trial registration have been strengthened. This evaluation builds upon a previous evaluation by similar authors, which found that one-quarter of 1017 RCTs approved between 2000-2003 were discontinued prematurely; 44% remained unpublished at a median of 12 years follow-up. In the current evaluation, which included 327 RCTs protocols approved in 2012 by research ethics committees in Switzerland, UK, Germany, and Canada, the authors found that 31% were discontinued prematurely; 24% remained unpunished at 9 years follow-up.

Overall, the authors conclude the premature trial discontinuation has not improved in the past decade. Although non-publication of RCTs has improved, opportunities exist for improvement. Overall, this is an important manuscript, which builds upon a prior, seminal evaluation. The findings remain relevant to a broad clinical audience.

**Response**: We thank reviewer 3 for the positive feedback and for highlighting the importance of our results.

Here are a number of suggestions for the authors and editors to consider:

1) First, as the authors note, the sample size is rather small. In particular, the sample is nearly a third of the size of the previous evaluation. In the published protocol, the authors note that they identified 450 protocols approved in 2012 and 402 protocols approved in 2016. Is there a reason that the 2016 protocols were not considered? In particular, the authors note that they "propose to compare RCT cohorts from 2012 and 2016 with RCTs approved 2000-2003…". If the authors have these data collected, a larger sample size would also strengthen the logistic regression-based analyses conducted, which may have been underpowered.

**Response**: As Reviewer 3 stated above, we have conducted a 9-year follow-up of the study protocols that were approved in 2012 (with the current update of the search now actually a 10-year follow-up). To have a meaningful and fair comparison, also the protocols that were approved in 2016 should have a comparable/sufficiently long follow-up time. Hence, we will conduct a study with protocols from 2016 at a later time point.

2) Second, the authors may want to consider updating the discussion section to include a brief section with potential implications. Currently, the authors recap the main findings (paragraph 1), provide a comparison with other studies (paragraphs 2 and 3), and present strengths and limitations. However, opportunities exist to mention several implications and/or recommendations, given that there has been little improvement over the past two decades.

**Response**: We agree and have, therefore, added a paragraph labelled “Implications” in the Discussion (see revised manuscript, line 427-445).

3) Third, it appears as if new data are reported in the Discussion section that are not included in the Results section (e.g., Supplementary Table 5). In particular, the authors present a table comparing study-protocols approved in 2012 with study-protocols approved in 2000-2003 in the Discussion section (data from the previous publication). As the authors note in the protocol, one of their objectives was to "compare the RCT cohorts from 2012 and 2016 with RCTs approved 2000-2003". However, these comparisons are not described in the Method or reported in the Results of the manuscript. To improve clarity, the authors could consider updating the Results section to include information from the formal comparison between the two samples, especially if they hope to discuss these data in the Discussion section.

**Response**: We agree and have included these results, as also suggested by the editors, in the Results section of our manuscript (see revised manuscript, lines 342-345).

4) Lastly, some of the results from the reported multivariable analyses may be difficult to interpret. In particular, those focused on odds ratios based on increments of 10% proportion of adherence. Why were increments of 10% proportion adherence selected? ("guidelines were less likely to not published their results in a peer-reviewed journal (OR 0.73 (in increments of 10% proportion adherence); 95%CI: 0.57-0.93)). This feel rather arbitrary, especially given that the SPIRIT checklist has 33 items.

**Response**: We agree that there are different ways of presenting this and we were also considering presenting this result in improvement of additional items reported. However, we believe that several readers are not familiar with the SPIRIT reporting guidelines; hence we have chosen to present this as “increments of 10% proportion adherence”. As also the statistical reviewer has not commented on this point, we would prefer keeping the current presentation of the results.

Specific comments:

Page 5, Abstract:

5) Line 139: Overall, the abstract is very clear and concise. The odds ratio (0.73 for each 10% increment in the proportion of SPIRIT items met) was the only part that may need some refining (please see above).

**Response**: Please see our answer to comment 4 from Reviewer 3 above.

6) Line 141: Perhaps the authors could rephrase "Non-publication of RCTs has improved" to increase clarity. For instance, the proportion of RCTs that remain unpublished has decreased. I think the "non-publication" and has "improved" makes it challenging to identify whether it is good or bad.

**Response**: Thank you for pointing this out. Following also the suggestion from reviewer 2 (see comment 5 from reviewer 2), we have rephrased the sentence to “*Non-publication of RCTs has declined,…*” (see revised manuscript, lines 156-157).

Introduction:

7) Line 150: The authors note that "A number of studies have explored the proportion of published RCTs that are registered (5-8); however, such investigations use published RCTs - and not RCT protocols - as the denominator and thus unable to assess publication bias." However, it is worth noting that previous studies have also looked at the proportion of registered RCTs that are published (e.g., <https://www.bmj.com/content/344/bmj.d7292> ; <https://jamanetwork.com/journals/jama/fullarticle/1840223> ; <https://journals.plos.org/plosmedicine/article?id=10.1371/journal.pmed.1000144> ; etc. [disclosure: I am not a co-author on any of these manuscripts]. These studies assess publication bias, so it is worth mentioning these efforts and the corresponding results.

**Response**: We agree that the mentioned studies are highly relevant when assessing publication bias. However, the first paragraph of the introduction summarizes what has already been done in terms of assessing what proportion of trials are registered. Hence, we would prefer not including the proposed references.

Methods:

8) Line 166: It was great to see the authors transparently report that their analysis of discontinuation due to preventable reasons was not prespecified. What prompted the additional analysis? Perhaps, the authors could include a short paragraph (supplementary materials?) outlining any changes from the published protocol?

**Response**: Thank you for acknowledging that we report which analyses were pre-specified. We have described now all changes from the protocol more consistently within the manuscript’s “analysis” section (see revised manuscript line 283-297). Furthermore, we have added a statement why the variable “discontinuation due to preventable reasons” was added. As pointed out by reviewer 2, discontinuation may not always be a bad thing (e.g. discontinuation for efficacy; see revised manuscript line 280-283).

9) Line 191: I may have missed this, but how were the trials matched if they did not have the same title, abbreviation, etc?

**Response**: Trials were matched using the information from titles, short titles, available registry numbers (as extracted from study protocols), and by considering the study population, intervention and control group. We also used the last name of the principal investigator to confirm matching. This procedure is described in the manuscript (lines 251-254) and in the Appendix 1.

Results:

10) Line 227+: How did these characteristics compare to the previous sample? Do the authors have information about therapeutic areas?

**Response**: We have added the type of intervention and therapeutic areas to the revised Table 1 and have added a sentence in the Results section, highlighting that characteristics of the two samples are comparable (see revised manuscript, Table 1, line 317-318).

11) I may have missed this in the Results, but did the authors report the following in the text:

- How many trials were prematurely discontinued were not published?

**Response**: This information is available in Appendix Table 3, and Table 3. We have now added also in the results section a sentence “*From the discontinued RCTs 67% (66/98) were available as a peer reviewed publication*” (see revised manuscript, lines 337-338).

- The proportion of trials that remained unpublished at 9 years follow-up? This shows up in the Discussion, but I did not see it in the Results text.

**Response**: Our manuscript states the proportion of results that were available in a registry (“*Approximately half (53%; 173/326) of all RCTs reported their results in a trial registry (industry-sponsored 84% [150/179] vs. 16% [23/147] investigator-sponsored)*…”) and as a peer reviewed publication (“…and 79% (256/326) of trials were published in a peer-reviewed journal.”). In addition, we also specified of how many RCTs no results were available (“*Results for 42 RCTs (13%) were neither published nor reported in a trial registry.*”). Please see revised manuscript, lines 324-326 and 330-331.

- The proportion of prospectively registered RCTs (83%). This shows up in the discussion but I did not see it in the Results text.

**Response**: The information is available in the Results section “*Of 327 RCTs, 94% (307/327) were registered (84% prospectively; 273/327, 10% retrospectively; 34/327), and 6% (20/327) of trials were unregistered (Table 2)*” (see revised manuscript, lines 320-322).

Discussion:

12) The authors note that "of the 20 RCTs we classified as unregistered, the status of 11 was unclear". Was this reported in the Results text?

**Response**: We apologize that this was not reported in the Results section. We have now added this information to the Results in the revised manuscript (line 320-322).

Figures/Tables:

13) Table 3: Perhaps the authors could stratify the rows in the table, since all the rows before the last one add up to 100%.

**Response**: Only the rows in the columns “All Discontinued RCTs”, “Industry-sponsored discontinued RCTs”, and “Investigator-sponsored discontinued RCTs” would add up to 100% when “Discontinuation due to a preventable reason” is presented separately. The rows of other columns would not add up to 100%. Since we introduced the category of “preventable reasons” only post-hoc (not specified in our published protocol) we would prefer keeping the Table as is. If we structured the Table by a stratification “preventable” and “non-preventable reasons”, this stratification would get more weight/attention and would seem planned from the start. .

**Reviewer #4:**

Many thanks for the opportunity to review this manuscript. Overall, this seems to be a very methodologically solid piece of work, meets the stated aims, and aligns with the published protocol. I also commend the authors on being clear about deviations throughout their manuscript. My comments are generally around minor clarifications and elaborations.

**Response**: We thank reviewer 4 for the positive feedback.

Abstract:

1) Page 6, lines 135-136: "We recorded whether RCTs were registered, discontinued (including reason for discontinuation), or published." Based on my understanding, this should say "and published" not "or published."

**Response**: We agree with the reviewer and changed the wording to “and published” (see revised manuscript, lines 137-138).

Introduction:

2) I'd like to see a little bit more discussion of the overall ASPIRE project and the role of this study within the larger project. No need for excruciating detail, as I know you clearly reference the overall project protocol, but the context is useful for readers to understand where this fits into the larger project and why the work was undertaken.

**Response**: Thank you for this suggestion. We added the following information to the beginning of the Methods section to provide readers with context of the overall ASPIRE project:

*“The present study is an associated project of the Adherence to SPIrit REcommendations (ASPIRE) study [2]. The ASPIRE study group is an international collaboration of researchers with a mandate to evaluate the completeness of RCT protocols before and after publication of the Standard Protocol Items: Recommendations for Interventional Trials (SPIRIT) statement (Gryaznov et al., BMJ Open, in press). Further ASPIRE sub-studies examine the use of patient-reported outcomes in RCT protocols, the reporting quality of RCT protocols with regulated vs non-regulated interventions, the planning of subgroup analyses in RCT protocols, and the use of routinely collected data in RCTs [2].”* (see revised manuscript, lines 217-223).

Methods:

3) Page 8 Line 189-193: Worth noting the ICTRP is not actually a registry but a meta-registry of entries from other registries (including the other ones you looked at). Also, I assume you actually used the EUCTR and not the back-end EudraCT system (which do not overlap perfectly as some things on EudraCT are not made public on the EUCTR).

**Response:** We apologize for the misleading wording in regard to ICTRP and EUCTR. We have now clarified that ICTRP is a Clinical Trial Registry Platform database and that EUCTR was searched (see revised manuscript, lines 244-250).

4) Why did you include the additional post-hoc independent variable? Seems like some reasoning or justification should be provided.

**Response**: We have described now all changes from the protocol more consistently within the manuscript’s “analysis” section (see revised manuscript line 283-297). We clarify which variables were pre-specified and which were included post-hoc following our previous conducted analysis in trial protocols approved between 2000-2003 to allow for a meaningful comparison (see also our reply to comment 8 of Reviewer 3).

5) Did you check for/account for duplicate registration?

**Response:** We documented if trials were registered multiple times. A trial was judged as registered if at least one registration was available and results were deemed as available in a trial registry if at least one trial registry contained results. With respect to multiple registration, we kindly refer the reviewer to a recently published separate analysis from our group entitled “*Reliability of Trial Information Across Registries for Trials With Multiple Registrations: A Systematic Review*” [3].

6) Can the authors please note their data sharing plans in the manuscript? While some information from the RECs may not be able to be shared, it seems to me that the overall dataset, or at the very least any analytic code, should be able to be shared. I strongly encourage the authors to do so or explain why they are not sharing their code and data.

**Response:** Thank you for raising this point. We conducted this study in close collaboration with research ethics committees and have written agreements with them. We are happy to share our analytical code and revised our Data Availability Statement as follows:

*“All participating ethics committees were project partners and granted us access to confidential study protocols under the condition that only aggregated data will be made publicly available. All investigators signed a corresponding confidentiality agreement with ethics committees. All aggregated data that were used for this study are presented in Tables and Figures of the manuscript or the appendix. The appendix also includes the used statistical code (STATA; Appendix 4).”* (see revised manuscript line 472-477).

In case the reviewer or the editors have any suggestions how we can better resolve this issue, we are happy to consider these.

Results:

7) One cut of the results I think is clearly missing is how many results were unique to a registry, especially among discontinued trials. Since publication in a peer reviewed journal might at times be difficult for a prematurely ended study, this is where registries really have the ability to shine as a dissemination mechanism. It seems you have the ability to offer some data here and I think it would be valuable to present.

**Response:** The reviewer raises an important point. We have therefore added this information within Table 2 and in the Results section (see revised manuscript, line 332-334). Results stratified for completed and discontinued RCTs were now added to Appendix Table 3.

8) Since only 30.3% of your sample actually reported a recruitment projection in the protocol (per Table 1), how did you then use this as an independent variable in your regressions? Seems like you would have had a lot of missing data to contend with and don't describe this anywhere. Perhaps I'm misunderstanding?

**Response:** We apologize that we missed to report this. We have now included in our Methods section, that reporting (or not reporting) of a recruitment projection was included in the regression model (see revised manuscript, line 283-289).

Discussion:

9) The context of this paper in relation to existing work is very sparse. You cite a bit about registration and then compare your findings to your previous findings. I would like to see a bit more depth in terms of delving into the existing literature related to your findings, for instance on reporting of industry vs. non-industry findings. In addition, I don't see a citation of Schmucker et al. 2014 anywhere (<https://pubmed.ncbi.nlm.nih.gov/25536072/>) which seems a very important citation as it is, to my knowledge, the most recent systematic review that includes an analysis of studies of eventual publication of trials approved by RECs.

**Response:** We agree and also thank the reviewer for making us aware of the study by Schmucker and colleagues. We have implemented this reference in our discussion and also state that the two identified studies by Schmucker et al found even lower publication rates for RCT study protocols that received ethical approval between 1988-1998 (see revised manuscript, lines 398-403). Furthermore, we have underlined that industry-sponsored studies often perform better when assessed in similar meta-research studies (see revised manuscript, lines 394-398).

10) I'd also like to see some more engagement with the implications of these findings in the Discussion. The whole section feels a bit empty. I'm left, as a reader, with little indication as to what these findings mean for the larger clinical trial landscape and what changes the authors might like to see or recommend or reflect on what has been done. The most we get is a single sentence suggesting more feasibility studies.

**Response:** We thank the reviewer for raising this point. We have now added an “Implications” paragraph to the Discussion section (see revised manuscript, line 427-445).

11) Another potential limitation is your use of the SPIRIT checklist in a way that assumes equivalence of the points. Additionally, there have been objections raised to using checklists as indicators of quality as the authors have done so here (<https://onlinelibrary.wiley.com/doi/full/10.1002/hsr2.165>).

**Response:** Thank you for the opportunity to clarify this point. We fully agree with the reviewer that reporting guidelines were typically not designed to be used as measurement tools for reporting quality. We carefully operationalized the SPIRIT guideline so that we were able to apply it in a standardized manner to included RCT protocols. We then conducted several analyses assuming different weights for different categories of SPIRIT items. This is described in detail in our protocol paper (Gryaznov et al. Trials 2020 [2]). The results of the main analysis of the ASPIRE (Adherence to SPIrit REcommendations) study showed (among other findings) that all our different assumptions with respect to weighting of SPIRIT items led to very similar estimates of reporting quality (Gryaznov et al BMJ open 2022, *in press*). We still added the reviewer’s point to our limitation section as follows:

*“Third, the SPIRIT checklist was created as a reporting guideline and not as a measurement tool for reporting quality [24]. However, we carefully operationalized the SPIRIT checklist and conducted various sensitivity analyses before using reporting quality estimates for the present study [13, 14].”* (see revised manuscript, line 417-420).

Minor Comments:

12) Page 8 Line 181: "Pairs of reviewers determined, independently and in duplicate" redundant to say pairs worked in duplicate.

**Response:** We agree; following also the suggestions from reviewer 1 (comment 7) we have rephrased the sentence (see revised manuscript, line 238-240).

13) Just to make the authors aware there are still some track changes in the supplement.

**Response:** We thank the reviewer for noticing this and have now removed all track changes from the appendix (see revised appendix).

**References**

1.	Speich B, Schur N, Gryaznov D, von Niederhäusern B, Hemkens LG, Schandelmaier S, et al. Resource use, costs, and approval times for planning and preparing a randomized clinical trial before and after the implementation of the new Swiss human research legislation. PLoS One. 2019;14(1):e0210669. Epub 2019/01/12. doi: 10.1371/journal.pone.0210669. PubMed PMID: 30633776; PubMed Central PMCID: PMCPMC6329511 study (Swiss Federal Office of Public Health) has implemented the new law on research with human beings (Human Research Act, HRA) and its ordinances (Swiss Legislation on Human Research, LHR) which is evaluated within this study. This does not alter our adherence to PLOS ONE policies on sharing data and materials.

2.	Gryaznov D, Odutayo A, von Niederhausern B, Speich B, Kasenda B, Ojeda-Ruiz E, et al. Rationale and design of repeated cross-sectional studies to evaluate the reporting quality of trial protocols: the Adherence to SPIrit REcommendations (ASPIRE) study and associated projects. Trials. 2020;21(1):896. Epub 2020/10/30. doi: 10.1186/s13063-020-04808-y. PubMed PMID: 33115541; PubMed Central PMCID: PMCPMC7594472.

3.	Speich B, Gloy VL, Klatte K, Gryaznov D, Taji Heravi A, Ghosh N, et al. Reliability of Trial Information Across Registries for Trials With Multiple Registrations: A Systematic Review. JAMA Netw Open. 2021;4(11):e2128898. Epub 2021/11/02. doi: 10.1001/jamanetworkopen.2021.28898. PubMed PMID: 34724557; PubMed Central PMCID: PMCPMC8561329.
